# The survey of prevention of accidents in mothers with children under the age of 5 years

**Published:** 2019-08

**Authors:** Maryam Fathi Sheikhi, Mohsen Shamsi, Mahboobeh Khorsandi, Raheleh Soltani

**Affiliations:** ^*a*^Department of health education, School of Health, Arak University of Medical Sciences, Arak, Iran.

## Abstract

**Background:**

All incidents are predictable and preventable. Therefore, the mother, as the first child care provider, while being aware of the treatment of complications from accidents needs to know the ways of preventing accidents. Purpose: The aim of this study is determine the prevention behaviors of accidents in mothers with children under the age of 5 years.

**Methods:**

This analytical study was carried out on 261 mothers with children under the age of 5 years referred to health centers in Khorramabad. The data collection method was demographic variables and preventive behaviors for accidents in children. Data were analyzed by SPSS software version 20.

**Results:**

The mean and standard deviation of the age of children was 21.13± 16.55 months and the mean and standard deviation of mother’s age was 28.98 ± 5.37 years. The mean score of mothers performance was 59.1 ± 14.74 (out of 100 score).

**Conclusions:**

In this study, the performance of mothers was about 59.1 out of 100, which, in view of this function of mothers, resulted in accidents and accidents and the referral of children with acute problems in this area to hospitals. Therefore, according to the results of this study, the design and performance an educational program for mothers as the first child care necessary.

**Keywords:**

Prevention, Accidents, Mother, Child

